# Systemic anti-commensal response to fungi analyzed by flow cytometry is related to gut mycobiome ecology

**DOI:** 10.1186/s40168-020-00924-8

**Published:** 2020-11-15

**Authors:** Alicia Moreno-Sabater, Gaelle Autaa, Delphine Sterlin, Amenie Jerbi, Remy Villette, Johanna B. Holm, Christophe Parizot, Sameh Selim, Yaye Senghor, Pascale Ghillani-Dalbin, Claude Bachmeyer, Christophe Hennequin, Guy Gorochov, Martin Larsen

**Affiliations:** 1 Sorbonne Université, Inserm U1135, Centre d’Immunologie et des Maladies Infectieuses (CIMI-Paris), 75013 Paris, France; 2 Service de Parasitologie-Mycologie AP-HP, Hôpital Saint-Antoine, 75012 Paris, France; 3 Service d’immunologie, AP-HP, Hôpital Pitié-Salpêtrière, 75013 Paris, France; 4 Unit of Antibodies in Therapy and Pathology, Institut Pasteur, UMR1222 Inserm, 75015 Paris, France; 5 Institute for Genome Sciences and Department of Microbiology and Immunology, University of Maryland School of Medicine, Baltimore, MD USA; 6 College of Agricultural Sciences AGHYLE Res, Unit. Institut Polytechnique UniLaSalle, 60026 Beauvais, France; 7 AP-HP, Hôpital Tenon 4, rue de la Chine, 75020 Paris, France; 8 Centre de Recherche Saint-Antoine, CRSA, AP-HP, Sorbonne Université, Inserm, 75012 Paris, France

**Keywords:** Mycobiota, Flow cytometry, Systemic anti-commensal responses, Humoral immunity, Immunoglobulin G, ITS rRNA gene sequencing

## Abstract

**Background:**

Interest for the study of gut mycobiota in relation with human health and immune homeostasis has increased in the last years. From this perspective, new tools to study the immune/fungal interface are warranted. Systemic humoral immune responses could reflect the dynamic relationships between gut mycobiota and immunity. Using a novel flow cytometry technology (Fungi-Flow) to determine immunoglobulin (Ig) responses to fungi, we studied the relationships between gut mycobiota and systemic humoral anti-commensal immunity.

**Results:**

The Fungi-Flow method allows a sensitive and specific measurement of systemic IgG responses against 17 commensal and environmental fungi from the two main divisions; *Ascomycota* and *Basidiomycota*. IgG responses exhibited a high inter-individual variability. Anti-commensal IgG responses were contrasted with the relative abundance, alpha-diversity, and intra-genus richness of fungal species in gut mycobiota of twenty healthy donors. Categorization of gut mycobiota composition revealed two differentiated fungal ecosystems. Significant difference of anti-*Saccharomyces* systemic IgG responses were observed in healthy donors stratified according to the fungal ecosystem colonizing their gut. A positive and significant correlation was observed between the variety of IgG responses against fungal commensals and intestinal alpha-diversity. At the level of intra-genus species richness, intense IgG responses were associated with a low intra-genus richness for known pathobionts, but not commensals.

**Conclusions:**

Fungi-Flow allows an easy and reliable measure of personalized humoral responses against commensal fungi. Combining sequencing technology with our novel Fungi-Flow immunological method, we propose that there are at least two defined ecosystems in the human gut mycobiome associated with systemic humoral responses. Fungi-Flow opens new opportunities to improve our knowledge about the impact of mycobiota in humoral anti-commensal immunity and homeostasis.

Video Abstract

## Background

Advances in high-throughput sequencing and bioinformatics are just beginning to reveal the breadth, depth, and diversity of human mycobiota [1, 2]. The gut mycobiome is receiving increased attention due to its potential involvement in the etiology of numerous gut-associated diseases. Intestinal fungal dysbiosis may be at the origin of invasive fungal infections due to *Candida* spp. [3] but it has also been associated with a number of gastrointestinal diseases, chemotherapy-induced enteric disorders, graft-versus-host disease [4], alcoholic hepatitis [5], neurological disease [6], and atopy [7] providing evidence of its ability to influence both local and distal inflammation.

The continuous interaction of the immune system with gut mycobiota is also recognized as important for immune homeostasis, playing a role in immune training, regulation of lymphocyte recirculation and immune tolerance [8–10]. These immunoregulation processes mainly occur at mucosal surfaces, thereby complicating the execution of human studies. Consequently, most of the current understanding of immune-mycobiota interactions, in health or disease, has been acquired from studies using animal models [11–17]. However, conclusions from animal models are hampered by the fact that mouse and human harbor distinct mycobiota ecosystems [18]. Although the use of an animal model is fundamental to decipher immunological mechanisms in situ, the development of new minimally invasive methods is mandatory to corroborate the relevance of this knowledge to human physiopathology.

In this context, flow cytometry methods have been developed to characterize human systemic and local immunoglobulin (Ig) responses to commensal bacteria [19]. This technology has allowed the study of whole bacteria and host Ig relationships from different biological fluids, providing a reliable measure of host exposure to a large range of bacterial species. Using this technology, it has been shown that there is a systemic and mucosal humoral response against commensal bacteria in healthy individuals [20]. Secretory IgA regulates gut microbiota compartmentalization, thereby protecting gut barrier integrity. Systemic anti-commensal IgG is implicated in protection against systemic microbial infections. Systemic anti-microbial IgG responses have also been associated with cancer and autoimmunity, although causality remains to be established in these settings. IgG and IgA have distinct biological roles, but seem to be related since human secretory IgA and systemic IgG converge to target a common fraction of gut microbiota [21].

There is now evidence that systemic anti-commensal responses to fungi play a role in promoting fungal clearance [22–24] and have been associated with immunopathologies characterized by intestinal fungal dysbiosis. Interestingly, high levels of anti-*Saccharomyces cerevisiae* antibodies (ASCA), a yeast highly present in the intestinal gut mycobiota, are associated with Crohn**’**s disease, antiphospholipid syndrome, systemic lupus erythematosus, type 1 diabetes mellitus, and rheumatoid arthritis [25]. These findings suggest a role of gut mycobiota in the modulation of anti-commensal systemic responses. However, presently, these responses have been characterized for a reduced number of fungi implicated in human pathology [26]. The main reason being the lack of high-throughput methods covering the high diversity of fungal exposition coming from human mycobiota. Consequently, it remains undiscovered which mycobiota members evoke host immune responses.

Here, we developed a flow cytometry technology for analyzing antibody responses to genotypically and phenotypically different fungi (Fungi-Flow) described in human mycobiota. We exploited the Fungi-Flow method to characterize IgG systemic responses to 17 commensal and environmental fungi in a healthy donor cohort. Combining flow cytometry analysis and mycobiota sequencing, we investigated the relationships between anti-commensal IgG and gut mycobiota diversity.

## Materials and methods

### Fungal biobank

A fungal biobank was generated including 17 fungal strains from different genera commonly described in human skin, lung, and intestinal mycobiota [27–30]. Fungal reference strains were obtained for *Candida albicans* (ATCC90028) and *Aspergillus fumigatus* (ATCC204305). *Kluyveromyces lactis*, *Debaryomyces hansenii*, *Yarrowia lipolytica*, *Saccharomyces cerevisiae*, *Fusarium oxysporum*, *Acremonium sclerotigenum*, *Cladosporium cladosporioides*, *Cyberlidnera fabianii*, *Cryptococcus neoformans*, *Penicillium oisonii*, *Rhodotorula mucilaginosa*, *Malassezia furfur*, *Trichosporon inkin*, and *Mycosphaerella graminicolla* and *Botrytis circinea* were obtained from in vitro culture of stools, skins, and bronchoalveolar lavages from human samples and vegetables. Except for *Cryptococcus neoformans*, the other fungal isolates belonged to the human flora or vegetables and were not implicated in human pathology. Fungal genus and species were determined by mass spectrometry analysis [31].

### Biological samples

To set up the Fungi-Flow method, IgG and IgA responses were measured in pooled IgG from healthy individuals (Hizentra®, CSL Behring), human serum from patients suffering from invasive fungal infections (*n* = 2), breast milk (*n* = 2), as well as in bronchoalveolar lavage (BAL) (*n* = 2). To show up the usefulness of the Fungi-Flow, a cohort of paired plasma and fresh stools (*n* = 20) were collected from healthy donors. Stool and plasma collection was carried out as previously described [21]. Oral and written consent were obtained from donors before inclusion in the study. The study protocol was reviewed and approved by the local ethics committee (Comité de Protection des Personnes Ile de France VI, Paris, France). All the biological samples were stored at − 20 °C until use except the stool samples which were stored at − 80 °C.

### Fungi culture conditions

Fungi were cultured in Sabouraud agar medium (Bio-Rad) between 30 and 35 °C, depending on the fungus studied. To generate budding forms, yeast or spores from filamentous fungi were kept at 4 °C in RPMI 1640 medium (Gibco) for 2 h and then cultured at 30 °C under stirring for 9 h. At the end of the incubation period, cultures were centrifuged at 21,000 g for 20 min. Supernatant was removed and fungi were resuspended in cryopreservation medium A or B. Cryopreservation medium A contains: 1% (wt/vol) Bacto Peptone (BD Biosciences), 1% (wt/vol) Bacto yeast extract (BD Biosciences), 0.5% Glucose (wt/vol) (Sigma Aldrich), and 25% Glycerol (vol/vol) (Sigma Aldrich) in distilled water. Cryopreservation medium B containing 10% of Glycerol (vol/vol) (Sigma Aldrich) in PBS (Gibco) was also evaluated. For buddingless fungi, only culture on solid Sabouraud agar medium was realized. Aliquots of 200 μl containing 10^4^–10^5^ fungal forms/μl were stored at − 80 °C until use.

### Quantification of fungi

To confirm the fungal origin of populations analyzed, fungal stock aliquots (200 μl containing 2.10^5^ fungal forms/μl) were thawed and incubated with 50 μl of calcofluor White staining (1 g/l, Sigma-Aldrich) during 15 min at 4 °C. Then, 400 μl of 2% (wt/vol) paraformaldehyde solution (ThermoFisher) was added to fix samples for 20 min at 4 °C. After 10-min 21,000 g centrifugation, supernatant was removed and pellet resuspended in the initial volume. Then, 25 μl of fungal suspension were mixed with 25 μl of flow count fluorospheres (Beckman Coulter) in 500 μl of PBS. Acquisition of microbeads and fungi was carried out using FACSCanto II flow cytometer (BD) and FACS Diva (BD) software.

### Analysis of sporulated and budding fungal forms

Sorting of *C*. *albicans* and *A*. *fumigatus* using a forward and side scatter variation gating strategy were performed on a 2-laser two-way fluorescence activated cell sorter (S3 cell sorter; Bio-Rad Laboratories, Hercules, California). The cell shape and purity for both fractions were verified by optical microscopy imaging (Leica DM2500).

### Fungal immunostaining detected by flow cytometry (Fungi-Flow)

Total Ig in serum samples were assessed by a nephelometry method (BN II, Siemens Health Care). In order to normalize the IgG or IgA concentration, serums were diluted to 20 μg/ml for IgG measure and 35 μg/ml for IgA measure in PBS (Gibco). Twenty-five microliters of fixed samples containing 2.5 × 10^5^ fungal forms were distributed in a 1.5 ml tube (Eppendorf). Twenty-five microliters of normalized serum were then distributed in each tube and incubated for 20 min at 4 °C. Two hundred microliters of PBS were then added and samples were centrifuged at 21,000 g during 10 min. Supernatant was removed and fungi were incubated with a 25 μl mix of goat anti-human IgG Alexa Fluor 647 (Jackson ImmunoResearch) or goat anti-human IgA FITC (Jackson Immunoresearch) or the isotype control, goat IgG A647/FITC (Jackson Immunoresearch), at 4 °C during 20 min. After one wash with PBS, stained fungi were resuspended in 200 μl of PBS. Acquisition of fungi was performed on a FACS Canto II flow cytometer (BD) using the FACS Diva (BD) software. Unstained spores or budding forms were used as negative control to identify background fluorescence. Monoclonal antibodies with irrelevant specificity (anti-TNF-α human IgG1; MSD laboratory) revealed Fc binding. Median fluorescence intensity (MFI) was used to assess the Ig response.

### DNA extraction from stool

DNA extraction was performed as previously described [21]. Briefly, 200 mg of fecal sample was lysed chemically (guanidine thiocyanate and N-lauroyl sarcosine) and mechanically (glass beads) followed by elimination of cell debris by centrifugation and precipitation of genomic DNA. Finally, genomic DNA was RNase treated. DNA concentration and molecular size were estimated by Nanodrop (Thermo Scientific) and agarose gel electrophoresis.

### Real-time quantitative PCR

16S or 18S ribosomal RNA gene levels were determined by real-time quantitative polymerase chain reaction (qPCR) using an ABI 7300 Real time PCR system (Applied Biosystems, Foster City, CA). Amplification and detection were carried out in 96-well plates and with TaqMan universal PCR 2X master mix (Applied Biosystems) to quantify total bacteria and fungi. Primers BactQuant and FungiQuant were used [32, 33]. Bacteria: forward: 5′-CCTACGGGDGGCWGCA-3′; reverse: 5′-GGACTACHVGGGTMTCTAATC-3′; probe: (6FAM) 5′-CAGCAGCCGCGGTA-3′ (MGBNFQ); Fungi: forward: 50-GGRAAACTCACCAGGTCCAG-3′; reverse: 50-GSWCTATCCCCAKCACGA-3; probe: (VIC) 50-TGGTGCATGGCCGTT-30 (MGBNFQ). Each reaction was done in duplicate in a final volume of 25 μl with 10 μl of appropriate dilutions of DNA sample (40 ng DNA). Amplifications were performed as follows: 95 °C for 10 min, to denature DNA and activate Ampli-Taq Gold polymerase, followed by 50 cycles of 95 °C for 15 s and 60 °C for 1 min. The threshold cycle for each sample was determined for 18S gene and normalized to the Ct value of the all bacteria 16S ribosomal RNA gene. Data were calculated using the 2-(2(-∆∆ Ct)) method [34].

### DNA sequencing

Sequencing libraries were constructed by amplifying the 18S rRNA gene ITS2 region using the PCR amplification protocol previously described [28]. DNA samples were subjected to PCR amplification using the following primers:

ITS2_FwTag (5′CTTTCCCTACACGACGCTCTTCCGATCT**GTGARTCATCGAATCTTT**-3′) and ITS2_RvTag (5′-GGAGTTCAGACGTGTGCTCTTCCGATCT**GATATGCTTAAGTTCAGCGGGT**-3′)

Amplification reaction was performed with DNA MolTaq (Molzym, Bremen, Germany) in a total volume of 50 μl containing 1 μM of each primer and 1 μl genomic DNA from extracted stool samples. Cycling conditions were initial denaturation at 94 °C for 60 s, 35 cycles of denaturation at 94 °C for 30 s, annealing at 56 °C for 30 s, and elongation at 72 °C for 45 s, followed by a final elongation step at 72 °C for 7 min. PCR amplicon libraries were finally sequenced on a MiSeq Illumina instrument (Genotoul, Toulouse, France) producing 2 × 300 bp paired-end reads.

### Gene abundance profiling

Further processing of demultiplexed sequence reads followed the DADA2 workflow for Big Data (https://benjjneb.github.io/dada2/bigdata.html) [35] and employed R software (version 3.5.3) and the DADA2 package (v. 1.5.2). Briefly, sequences were quality filtered, trimmed, and assembled. Read length of 240 bases was chosen for hard trimming of forward and reverse reads, because these were the lengths beyond which median quality scores decreased below 20 for the lowest-quality library. Then, 240 bases trimming length was also adopted to ensure that paired sequences contained sufficient information for merging (overlapping 3′ ends). Individual reads were truncated at the base, where a quality score of 2 was observed, and filtered to contain no ambiguous bases. Additionally, the maximum number of expected errors in a read was set to 2. Chimeras from combined runs were removed by the DADA2 protocol. Amplicon sequence variants (ASVs) generated by DADA2 analysis of the quality-filtered sequence data were taxonomically classified using the RDP naïve Bayesian classifier [35] trained with the UNITE general release v10.10.2017 ITS2 gene sequence database (https://unite.ut.ee/sh_files/sh_general_release_10.10.2017.zip). Read counts for ASVs assigned to the same taxonomy were summed for each sample. For the 20 healthy donor stool samples included in our study, we obtained 1,042,760 paired-end reads (average: 45,337 paired-end reads/sample), which was reduced to 583320 paired-end reads with appropriate phylogenetic assignment (average: 25,362 paired-end reads/sample). We finally rarefied our data to 11,872 paired-end reads per sample.

### Data analysis

Flow cytometry data analysis was performed using FlowJo program (version 9.3.2). Mycobiome composition was studied with the Phyloseq R package (version 1.24) and JMP Pro (version 14). Alpha-diversity was assessed with the Shannon diversity index. The distance matrix was subjected to agglomerative hierarchically clustering using Ward’s method. To assess the richness of anti-commensal IgG responses in our cohort, an index of IgG responses was determined using the Shannon index formula applied to the MFI values. Species and strain richness was measured as the number of different amplicon sequence variants (ASV) in a genus. Statistical analysis was performed with GraphPad Prism® (version 6) and JMP Pro (version 14).

## Results

### Flow cytometry analysis differentiate sporulated and budding forms of fungi

**Fig. 1:**
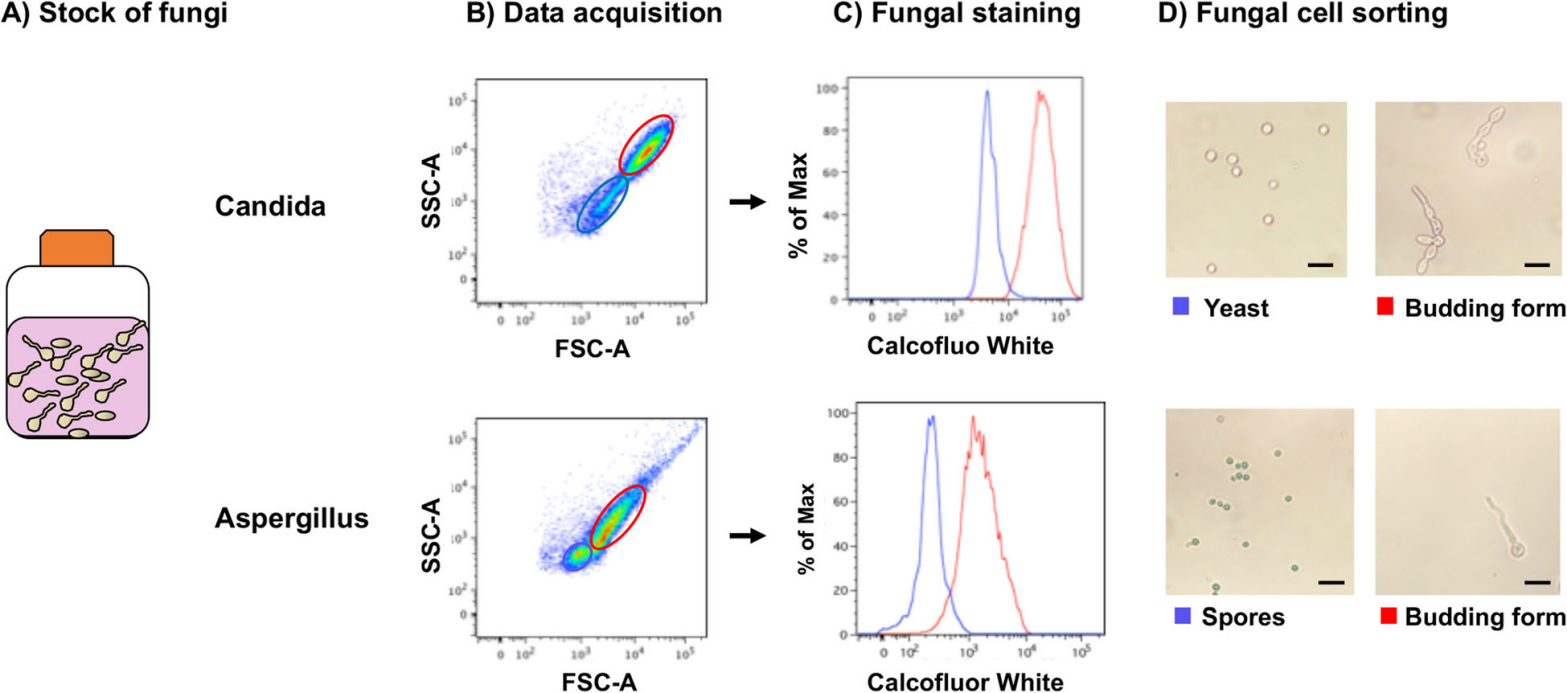
Fungi-Flow discriminates between sporulated and budding fungal forms. **a** Overnight culture of sporulated forms in RPMI medium. **b** Acquisition by flow cytometry permits discrimination of two populations according to size (FSC) and granularity (SSC). **c** Calcofluor White staining of both fungal populations. **d** Fluorescence-Activated Cell Sorting of two *C*. *albicans* and *A*. *fumigatus* populations shows a yeast or spores enriched population, which is well differentiated from the budding form population (Light microscopy imaging × 400).

A striking feature of fungal biology is its large heterogeneity. Fungi can be yeast or molds and throughout their life cycle they have the ability to adopt different phenotypes, such as sporulated, pseudohyphal, or hyphal forms. Furthermore, antigens differ between different phenotypes [36]. In order to ensure that flow cytometry technology is able to cover a large range of fungal antigens and ensure the assessment of the whole fungal antibody repertoire from sporulated and buddings forms, we produced overnight cultures of *C*. *albicans* and *A*. *fumigatus* (Fig. 1).

Forward and side scatter dot plots allowed differentiating two populations based on cellular size and structure. Both these populations bound Calcofluor White, which is a fluorescent blue dye binding to polysaccharides on chitin and cellulose that are present in cell walls on fungi. Using Fluorescence-Activated Cell Sorting technology, both populations were separated and subsequently analyzed by microscopy. The population characterized by low SSC-A/FSC-A measures corresponded to non-budding yeast for *C*. *albicans* or spores for *A*. *fumigatus*, whereas the population with high SSC-A/FSC-A measures corresponded to budding forms of *C*. *albicans* or *A*. *fumigatus*.

To ensure reproducibility, we sought to produce batches of fungus of a sufficient size to analyze all biospecimens of a given series or cohort. To determine optimal in vitro culture conditions for producing batches of fungal sporulated and budding forms, dose response curves were performed using different initial concentrations of sporulated forms of *C*. *albicans* and *A fumigatus* (Additional file 1: Figure S1). Culture conditions tested for producing batches of *C*. *albicans* or *A*. *fumigatus* showed that low starting concentrations favored budding form formation. Using 5 × 10^6^ fungal forms/ml, we obtained both sporulated and budding forms for both fungi. Inoculation of 5 × 10^6^ yeasts or spores/ml is also suitable for *Fusarium* and *Acremonium* (data not shown).

### Validation and optimization of analytical specificity and sensitivity

**Fig. 2:**
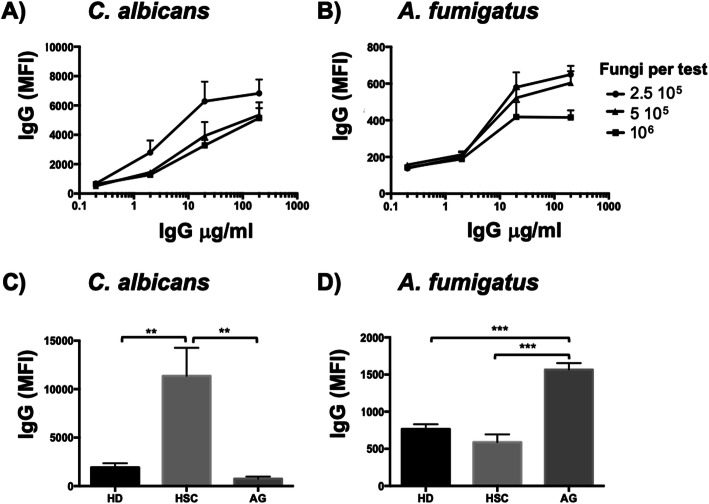
Fungi-Flow optimal conditions and specificity. **a**, **b** Dose response relationship was evaluated by plotting median fluorescence intensity (MFI) obtained for IgG responses at different combinations of *C*. *albicans* and *A*. *fumigatus* and serum antibody concentrations. Serum was obtained from patients with a confirmed hepatosplenic candidosis (HSC) or aspergilloma (AG). **c**, **d** Specificity of antibodies in sera from HSC, AG patients and healthy donor (HD). Results correspond to the mean and standard deviation (*n* = 3). Statistical analysis was performed using Student’s *t* test (**p < 0.05, ***p < 0.005)

We sought to set up Fungi-Flow method for anti-fungal IgG and IgA assessment. To determine the optimal IgG concentration to use in the Fungi-Flow method, we realized dose response curves with different fungal concentrations of *C*. *albicans* or *A*. *fumigatus* and sera from two patients with a confirmed hepatosplenic candidiasis or aspergilloma infection (Fig. 2a, b). Total IgG concentrations tested ranged from 0.1 to 1000 μg/ml. We observed the highest IgG-specific responses using 2.5 × 10^5^ fungi per test, for both fungi studied. At this optimal fungal concentration, serial dilutions of total IgG results in sigmoid curves reaching an upper plateau at 20 μg/ml. We therefore used serum dilutions of 20 μg/ml total IgG in combination with 2.5 × 10^5^ fungi per test for measuring specific IgG anti-fungal responses. The optimal IgA concentration was also assessed (Additional file 2: Figure S2). Dose response curves using 2.5 × 10^5^ fungi per test resulted in sigmoid curves of MFI reaching an upper plateau at 35 μg/ml.

In order to evaluate the specificity of the Fungi-Flow method, we evaluated the specific enrichment of IgG against *C*. *albicans* and *A*. *fumigatus* in sera from patients with hepatosplenic candidiasis, aspergilloma, and a healthy donor (Fig. 2c, d). IgG MFIs yielded by the hepatosplenic candidiasis serum against *C*. *albicans* were significantly higher than those corresponding to the aspergilloma or healthy donor sera (*p* < 0.05). In contrast, IgG MFIs yielded by the aspergilloma serum against *A*. *fumigatus* were significantly higher than those yielded by the hepatosplenic candidiasis or healthy donor sera (IgG: *p* < 0.005). Similar results were obtained when IgA specificity from these sera were evaluated against *C*. *albicans* and *A*. *fumigatus* (Additional file 2: Figure S2)*.* Moreover, using Fungi-Flow method, IgA could be detected in other biological samples such as breast milk and BAL.

We also evaluated the effect of fungal cryopreservation on Ig binding using two different preservation media, described in the materials and methods section (Additional file 3: Figure S3). Similar IgG binding to yeast or budding forms was observed whatever congelation medium was used demonstrating that cryopreservation does not alter Ig binding to fungi forms. We finally chose preservation medium A based on prior experience with clinical isolate preservation.

### Fungi-Flow overview

**Fig. 3:**
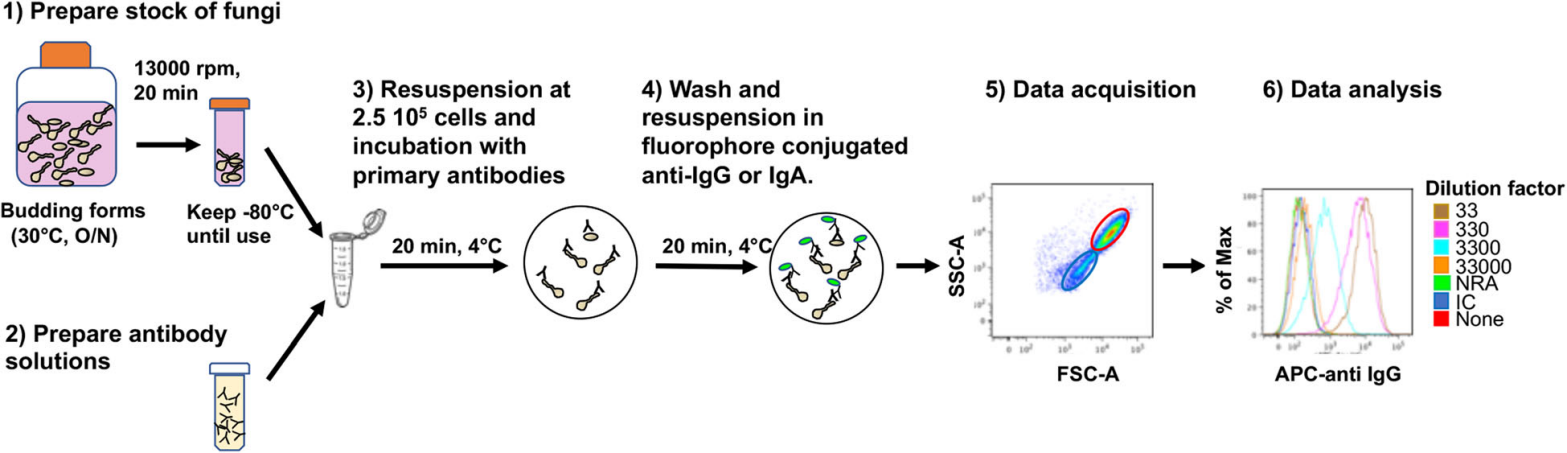
Overview of the Fungi-Flow protocol. Step 1: Fungal sporulated forms are cultured overnight at 30 °C to obtain budding forms. They were stored at − 80 °C until use. Step 2: Antibody samples are diluted to normalized concentrations. Step 3: Fungi are incubated with serum. Step 4: After washing, fungi are incubated with secondary antibodies. Step 5: Data acquisition. Acquisition by flow cytometry permits discrimination of fungi according to size (FSC-A) and granularity (SSC-A). Step 6: Data analysis. Histograms show the median fluorescence intensity (MFI) for yeast forms of *C*. *albicans* after incubation with different dilutions of serum from a healthy donor followed by immunostaining with fluorescently-labeled anti-human IgG antibodies. This protocol can be applied to any culturable fungi. Relevant negative controls must be used to detect unspecific fluorescence. None = unstained fungal forms. *NRA* non-relevant antibody, *IC* isotype control

After determining the optimal assay conditions, we can summarize the Fungi-Flow protocol as shown in Fig. 3*.* A frozen aliquot containing sporulated and budding fungal forms is thawed, fixed, and then incubated with human fluids to enable specific fungi-antibody interactions (steps 1–3). Then, antibody binding is detected by fluorescently labeled secondary antibodies specific for human immunoglobulins, such as IgG or IgA (step 4). Data acquisition is accomplished on a flow cytometer, which enables multiparametric phenotypic assessment, such as size and cellular complexity, biomarkers, such as DNA intercalating agents or chitin binding molecules (Calcofluor White) as well as antibody binding (step 5). Ig histograms are analyzed to determine the MFI of the fungal population and quantify the fungal specific antibody binding (step 6). The histogram shows the shift in anti-fungal IgG binding resulting from serial serum dilutions. Fungi-Flow should always be conducted using appropriate positive controls, e.g., immunized sera or polyclonal human IgG, and negative controls, such as no primary antibody, isotype controls, and irrelevant primary antibody (e.g., anti-TNF-alpha antibody).

### Systemic anti-commensal immunoglobulin response to fungi in healthy donor

**Fig. 4:**
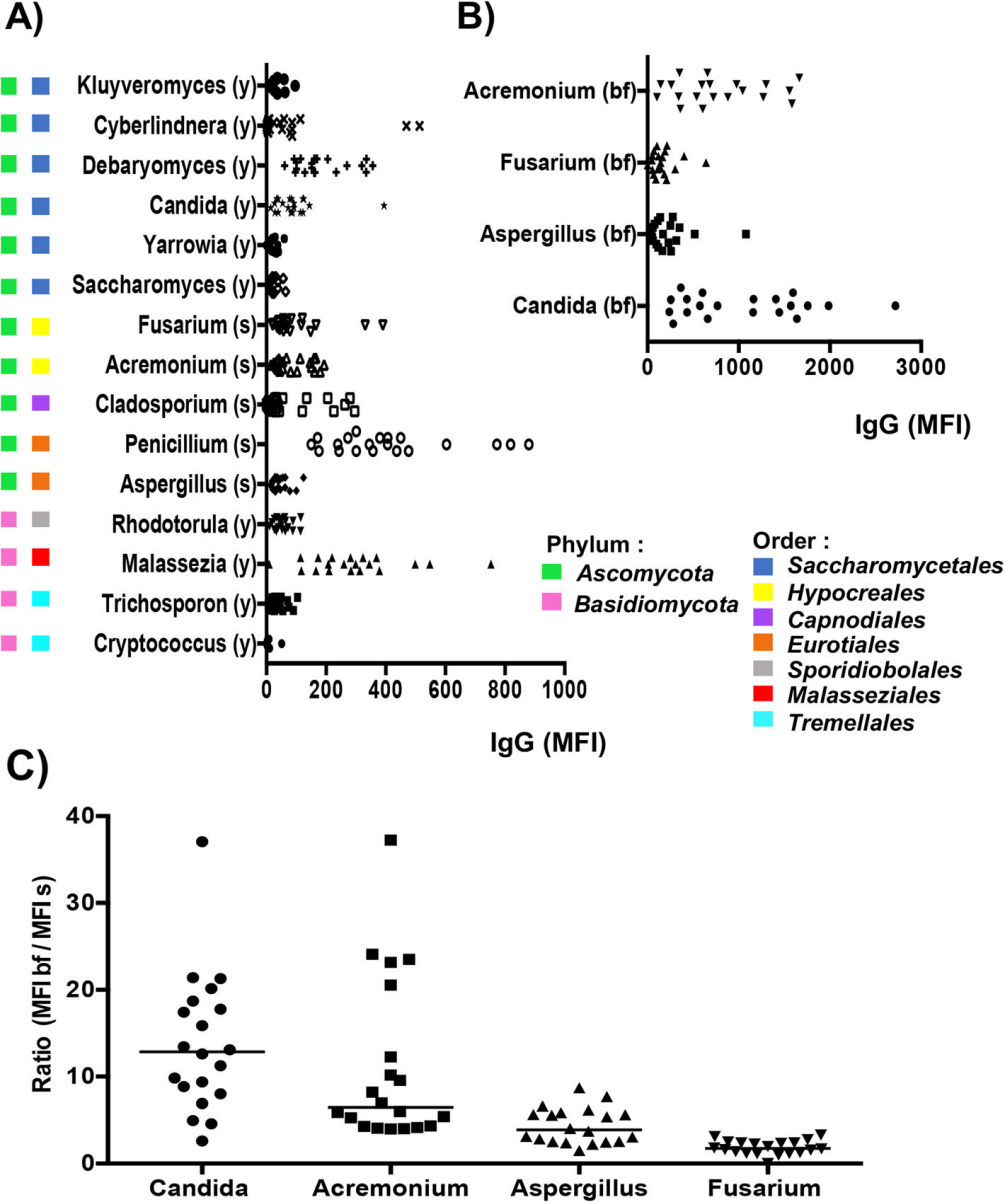
Fungi-Flow method allows an easy and reliable measure of human anti-commensal IgG responses. **a** Systemic IgG responses to 15 fungal genera, measured using the sporulated forms, are displayed as median fluorescence intensity (MFI) values and categorized according to phylogenetic relatedness. **b** Systemic IgG responses to 4 fungal general, measured using the budding forms. **c** Ratio between MFI IgG responses measured against budding forms versus sporulated forms of *Candida*, *Aspergillus*, *Acremonium*, and *Fusarium*. Results represent the mean of two independent assays. *y* yeast, *s* spore, *bf* budding form

We then evaluated the performances of the Fungi-Flow to determine the systemic anti-commensal IgG responses in 20 healthy donors. To cover the large fungal heterogeneity, 15 fungi from different genera of the two main divisions *Ascomycota* and *Basidiomycota* and commonly described in human skin, lung, and gut mycobiota were chosen [27–29, 37, 38] (Fig. 4). Specific IgG responses were measured against the sporulated forms (Fig. 4a) and for 4 of them equally against the budding forms (Fig. 4b). IgG responses exhibited a high inter-individual variability for a determined genus and this trend was observed for the fifteen different genera studied from the two main divisions. Similarly, anti-fungal IgG responses exhibited inter-taxonomic variability even for closely related taxons, e.g., in the *Saccharomycetales* order, IgG responses against *Candida* and *Debaryomyces* were significantly higher than those for *Saccharomyces* or *Kluyveromyces*. In the *Eurotiales* order, *Penicillium* showed higher responses than *Aspergillus*. In the *Basidiomycota* division, *Malassezia* from the *Malasseziales* order showed the highest IgG response, whereas *Cryptococcus* from the *Tremellales* order showed the lowest. Concerning the anti-commensal response against the budding and invasive fungal forms, the highest responses were observed for *Candida* and *Acremonium*, whereas the lowest were for *Fusarium* and *Aspergillus* (Fig. 4b). These results confirm that the Fungi-Flow allows an easy determination of systemic anti-commensal responses to a large panel of fungi.

Interestingly, we also noted differences in specific IgG responses between the sporulated or the budding form of a fungus (Fig. 4c). Serum IgG responses against the sporulated and budding forms were correlated but they were not proportional. Thus, for *Candida* and *Acremonium*, the IgG ratio between both forms ranged from 2 to 40, whereas the ratio for *Aspergillu*s and *Fusarium* ranged from 2 to 6.

### Systemic anti-commensal response to fungi is connected to gut mycobiota ecology

Intestinal mycobiota is an important source of fungal antigens that can elicit systemic IgG anti-commensal responses in animal models [16]. We therefore decided to study the relationships between gut mycobiome composition and the IgG systemic anti-commensal immunity determined using the Fungi-Flow method.

To this aim, global fungal load was determined by qPCR and we characterized the intestinal mycobiota in our healthy cohort of 20 individuals by ITS2 amplicon sequencing analysis. Phylogenetic entities, amplicon sequence variants (AVS), were identified and their relative abundance determined for each donor stool. The most abundant fungal genus in the gut mycobiota was *Saccharomyces*, followed by *Debaryomyces*, *Candida*, *Cyberlindnera*, and *Malassezia*, similar to previous observations in healthy individuals [38] (Additional file 4: Figure S4A).

Using the Fungi-Flow methods, IgG responses were analyzed for the 10 more abundant fungi (Additional file 4: Figure S4B). IgG responses for *Saccharomyces*, *Debaryomyces*, *Candida*, *Cyberlindnera*, *Malassezia*, *Penicillium*, and *Yarrowia* had been determined in the previous section. We then completed this analysis by evaluating the IgG responses against *Mycosphaerella*, *Botrytis*, and *Trametes*. While the IgG responses could be determined against the spore forms for the first two fungi, the lack of in vitro sporulation for the *Trametes* isolate prevented us from assessing the IgG response against this fungus. Thereafter, the correlation between the fungal relative abundance in gut and the intensity of the systemic response was evaluated. A significant correlation was observed for *Saccharomyces* (*r* = 0.51, *p* = 0.0201) suggesting that lower relative abundance of *Saccharomyces* in gut mycobiota is associated with an increase of ASCA responses.

**Fig. 5:**
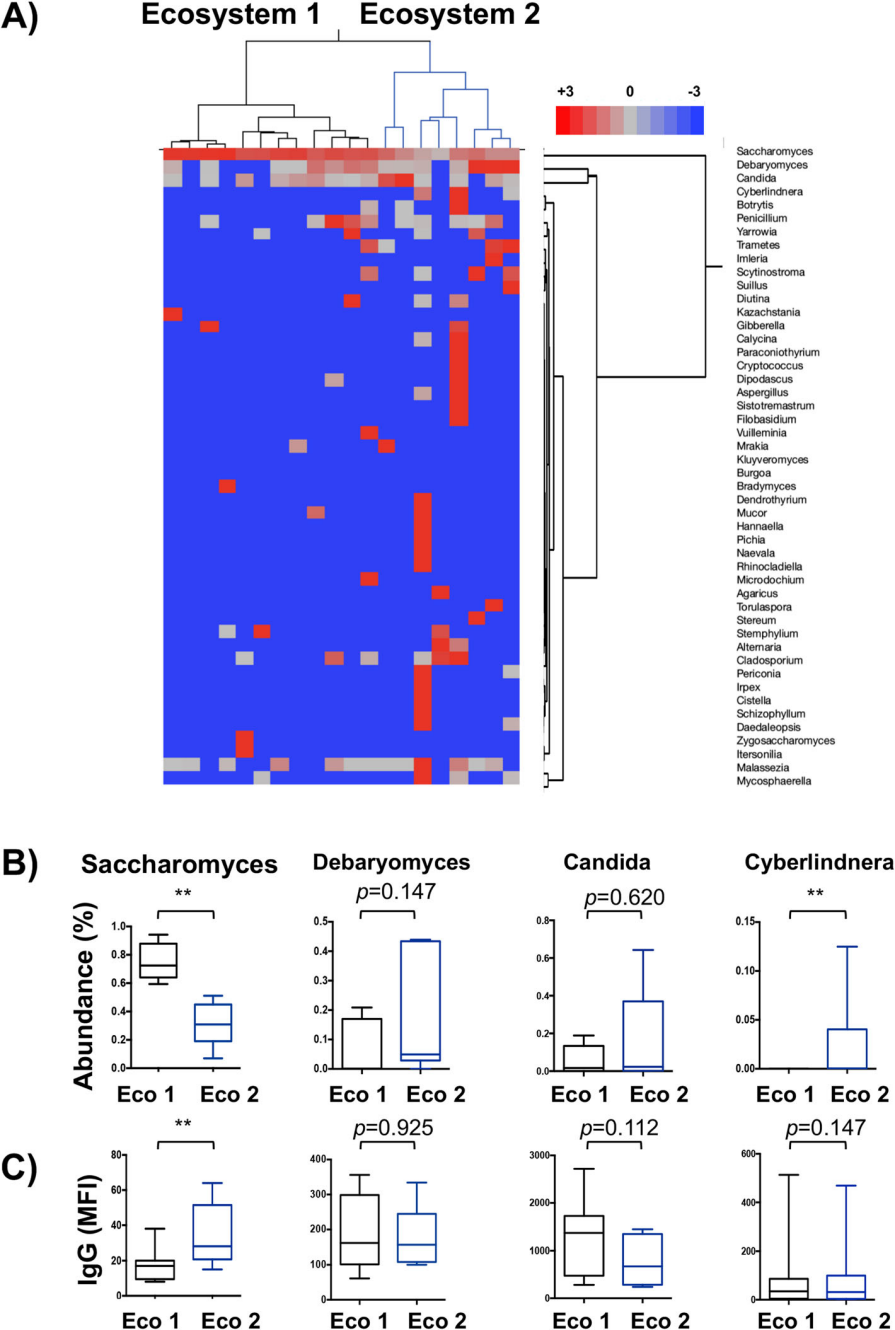
Gut fungal ecosystems in healthy donors have an impact in systemic anti-commensal response. **a** Hieratical clustering of the 50 most abundant species in gut mycobiome of healthy donors. This analysis revealed that our healthy population could be shared in two clusters showing two well-differentiated ecosystems (Eco). **b** Comparison of relative abundances between both ecosystems. Differences were mainly driven by the more abundant fungi, *Saccharomyces* but also by less abundant fungi such as *Cyberlindnera*. **c** Comparison of anti-commensal IgG responses between both ecosystems. Statistical analysis was performed using Mann-Whitney test (**p < 0.05)

We further conducted hierarchical clustering of our cohort using the relative abundance for the fifty more represented fungi (Fig. 5). Indeed, two clusters were obtained showing different intestinal fungal composition (Fig. 5a), which were named ecosystem 1 and 2. Compared with the ecosystem 1, ecosystem 2 was characterized by a significant decrease in *Saccharomyces* relative abundance and the presence of new genera, such as *Cyberlindnera* (Fig. 5b). No significant differences were observed in terms of fungal load between both groups (Additional file 5: Figure S5). It is presently unknown if colonization by a given ecosystems is associated with a particular systemic anti-commensal response. We therefore measured the IgG responses to fungi in healthy donors stratified according to their gut mycobiota ecosystem (Fig. 5c). Significantly different ASCA responses were observed between healthy donors harboring the ecosystem 1 and 2, with higher ASCA responses in healthy donors with lower *Saccharomyces* abundance (ecosystem 2). No correlation was observed between ASCA responses and the relative abundance or antibodies response to other studied fungi (data not shown).

### Systemic anti-commensal responses to fungi are related to gut mycobiota diversity

**Fig. 6:**
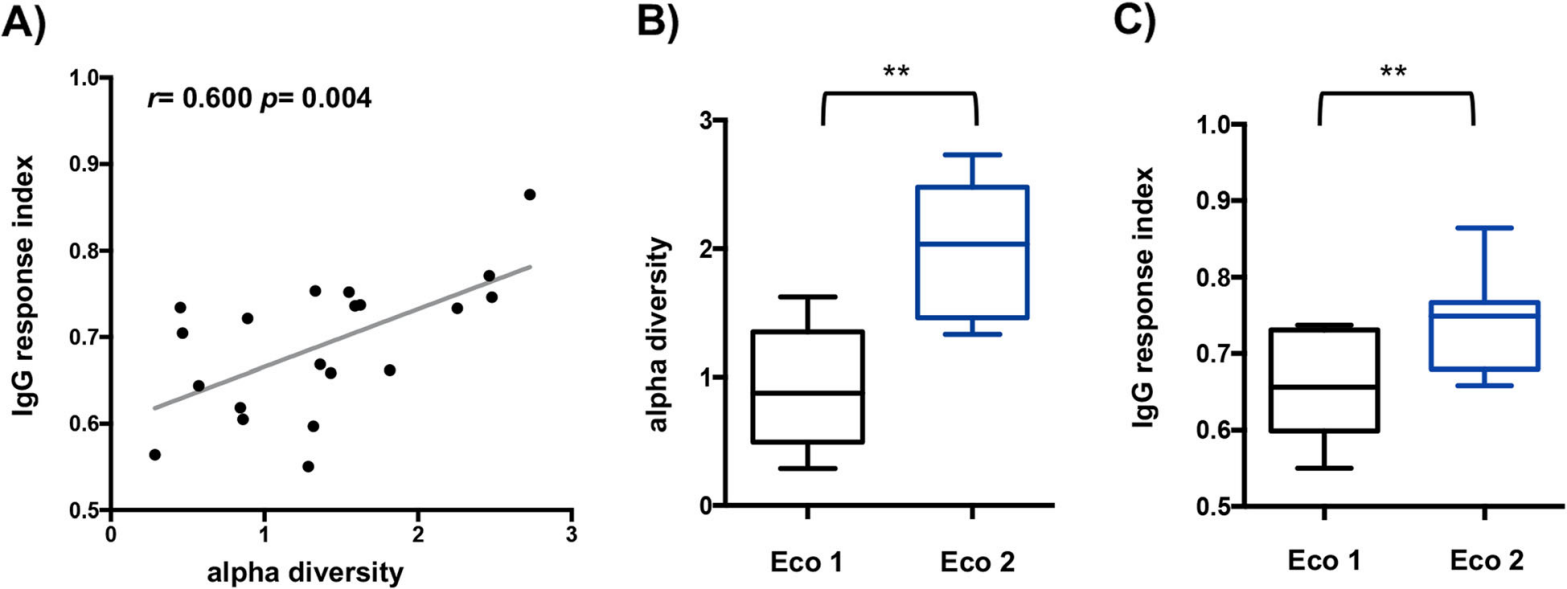
Diversity of the systemic anti-commensal IgG response is associated with alpha diversity of gut mycobiome in healthy donors. **a** Analysis of gut mycobiome diversity and its association with the IgG response index observed healthy donors. IgG response index was calculated using the Shannon index formula applied to the MFI values obtained for *Saccharomyces*, *Debaryomyces*, *Candida*, *Cyberlindnera*, *Malassezia*, *Mycosphaerella*, *Penicillium*, *Botrytis*, *Yarrowia*, *Cladosporium*, *Kluyveromyces*, *Cryptococcus*, and *Aspergillus*. **b**, **c** Analysis of alpha-diversity from gut mycobiome and IgG response index in healthy donors harboring different ecosystems. Statistical analysis was performed using Mann-Whitney test (**p < 0.05)

Subsequently, we hypothesized that gut mycobiota alpha diversity could impacts systemic responses to fungi. To explore this hypothesis, for each healthy donor, we determined an IgG response index using IgG response from fungi described in the previous section and those detected fungi in our healthy population mycobiota and available in our biobank (*Cladosporium*, *Kluyveromyces*, *Cryptococcus*, and *Aspergillus*) (Fig. 6). Consequently, IgG responses from *Fusarium*, *Acremonium*, *Rhodotorula*, and *Thrichosporon* were not included in this analysis.

The relationship between the IgG response index and the alpha-diversity of the intestinal mycobiome for each healthy donor showed a significant positive correlation, *r* = 0.61, *p* = 0.004. Further analysis revealed significant differences of both alpha diversity and anti-fungal immune diversity between the ecosystems 1 and 2 (Fig. 6b, c). Indeed, a low intestinal diversity was associated with low IgG response index in ecosystem 1, whereas higher diversity observed in the ecosystem 2 was associated with higher IgG response index.

### Systemic IgG responses are inversely associated with intra-genus gut richness

**Fig. 7:**
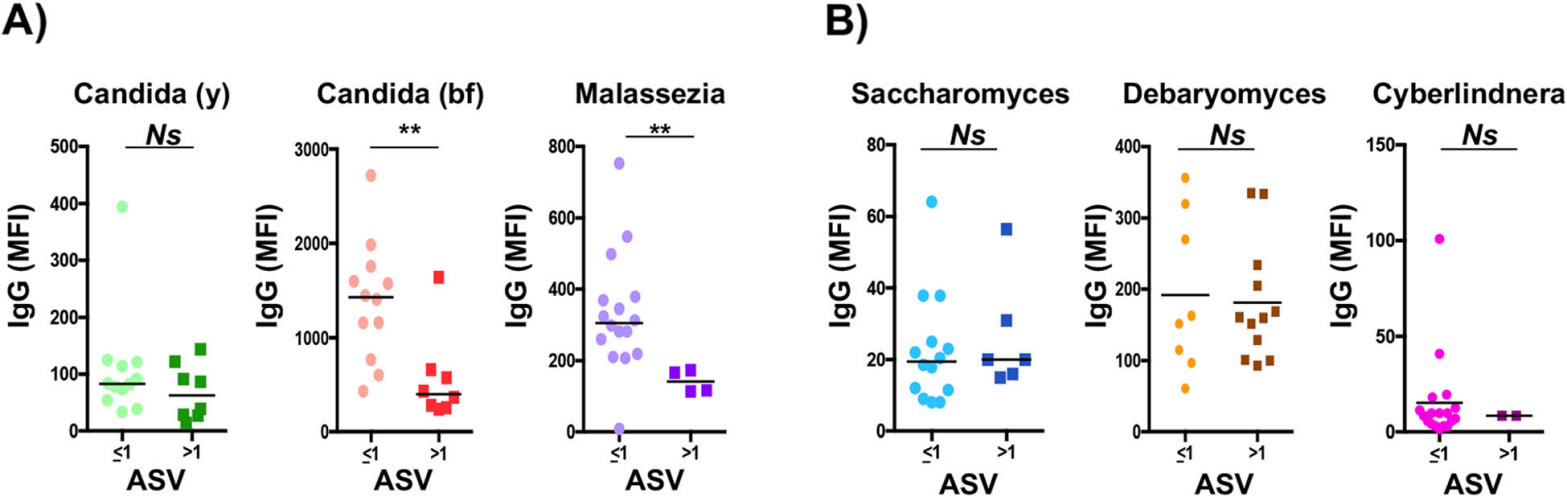
Relationship between systemic anti-commensal responses and intestinal species or strains richness. IgG response in donors harboring gut **a** pathobionts or **b** commensal fungi with less or more than one different amplicon sequence variant (ASV). Statistical analysis was performed using the Mann-Whitney Wilcoxon test (**p < 0.05). *Ns* non-significant difference

We also studied the relationship between systemic immunity and the intestinal intra-genus species or strains richness, measured as the number of different ASV belonging to a given genus (Fig. 7). We focused our analysis on two fungi with pathobiont properties, *Candida* and *Malassezia*, and three commensal fungi *Saccharomyces*, *Debaryomyces*, and *Cyberlindnera*. For *Candida*, the relationship between intra-genus richness and the intensity of the anti-commensal IgG responses to *Candida* was analyzed for the sporulated and budding forms. Systemic IgG responses against commensal pathobionts were significantly higher in those healthy donors having low intra-genus richness (Fig. 7a). Interestingly, for *Candida*, this difference was significantly different only when we analyzed the IgG response against the budding form, but not against the yeast form. In contrast, no significant difference was observed for the intensity of IgG responses when the intra-genus richness was analyzed for commensal fungi (Fig. 7b).

## Discussion

In this work, a new flow cytometry technology was developed to determine humoral responses against a large range of fungal genera covering the two main *Phyla*, *Ascomycota* and *Basidiomycota*. Our results shown that Fungi-Flow technology allows the assessment of systemic humoral response against 15 different fungal genera commonly described in human mycobiota and 2 specific fungal genera detected in the gut mycobiota of healthy donors included in this study. For 4 of those fungal genera, Fungi-Flow also allows the analysis of IgG responses against their sporulated and budding fungal forms increasing the pipeline of antigens analyzed. It also involves that Fungi-Flow technology is limited to spore forming fungus, which can be grown in vitro. Although not exhaustive, the 17 genera analyzed in the present study cover the most abundant fungal genera described in the human mycobiota [27–29, 37, 38]. This new methodology shows numerous advantages over ELISA and Western blot-based methods, due to its independence from intracellular antigens from lysed fungi and its ability to visualize fungal phenotypes. This technology requires minute quantities of serum and fungal targets thus allowing analysis of fastidious species.

Until now, the assessment of systemic humoral responses to commensal fungi has been hampered by their large variety and heterogeneity and the failure of isolating fungus from stool donors since the vast majority are refractory to cultivation or are rarely obtained in all human samples [1, 39–41]. To study the humoral immunity to commensal bacteria, incubation of healthy donor’s serum with isolated gut microbiota have been proposed [21]. However, due to the reduced burden of fungi as compared with bacteria in gut, this method mainly shows the impact of bacteria. As proposed in this study, the indirect assessment of systemic humoral response by incubation of healthy donor’s serum with a large variety of fungal strains using flow cytometry technology could move forward the study of immune system and mycobiota interaction as has been demonstrated for bacterial microbiota [19, 42].

Using the fungi-flow method, we have characterized for the first time the human anti-commensal IgG response under homeostatic conditions to a broad panel of fungi. IgG responses could be differentiated within a phylogenetic order or even at the genus level, showing a large range of responses inside a population. The intensity of IgG responses varied according to the fungi studied. Intense IgG responses were mainly observed against *Penicillium* and *Malassezia*. *Penicillium* is a common environmental fungus previously described in the lung and gut mycobiome [29, 38]. It is considered a commensal fungus but its presence in the human environment has been associated with asthma symptoms [43]. *Malassezia* though present in the intestinal mycobiome is also a skin commensal, involved in skin disorders [30]. A lower level of response was directed to fungi such as *Debaryomyces*, *Fusarium*, *Acremonium*, *Cladosporium*, and *Candida.* These fungi are largely represented in diet and environment and it is therefore less clear if they are colonizing commensals or simply in transit. The lowest anti-commensal responses were observed for *Kluyveromyces*, *Cyberlindnera*, *Yarrowia*, *Saccharomyces*, *Rhodotorula*, and *Trichosporon*. All these fungal genera are commensals from human gut or skin and have been rarely implicated in human invasive infections, which could explain the low intensity of anti-commensal response developed. Interestingly, very low IgG responses were detected for *Saccharomyces* despite high abundance of this fungus in the human gut mycobiome [38]. Altogether, our results show that Fungi-Flow allows the assessment of the anti-commensal IgG profile to a large variety of fungi revealing a fingerprint of fungal exposure in a healthy population.

Characterization of gut mycobiota composition of our cohort showed that healthy individuals can be attributed to one of two clusters representing two well differentiated ecosystems. Differences between the two ecosystems are mainly driven by *Saccharomyces* abundance and alpha-diversity. Combining the anti-commensal systemic humoral response with gut mycobiota abundance, we highlighted a significant and inverse correlation between the intensity of ASCA responses and *Saccharomyces* abundances in healthy donors’ gut. This observation provides a link between the gut ecosystems and the systemic response. The immunological mechanisms implicated in the ASCA response have not been elucidated. ASCA epitope is an oligomannose expressed in *Saccharomyces cerevisiae*, but also in other yeasts from the diet such as *Debaryomyces* [44] or *Kluyveromyces* [45] suggesting that diet can be a source of antigenic stimulation. ASCA epitope is also expressed in *C*. *albicans*, highly present in human gut mycobiota [46]. Fungal dysbiosis with an increase in *Candida* relative abundance has been associated with ASCA response in different human pathologies [5, 28]. Our study associates for the first time ASCA with gut fungal ecosystem in homeostatic conditions, for which fungal burden did not differ. No correlation was observed between ASCA response and yeast abundance, other than *Saccharomyces*, suggesting that others factors play a role in the antigenic stimulation of the ASCA responses observed in a healthy population.

Analysis of mycobiota alpha-diversity and the variability of IgG response revealed a positive and significant correlation suggesting that not only *Saccharomyces* genus but also the remaining studied fungal genera detected in the mycobiota of our healthy donor have an impact on the anti-commensal response. Indeed, healthy donors harboring a highly diverse ecosystem 2 developed anti-commensal systemic responses significantly different to those observed in healthy donors harboring a lower diverse ecosystem. Finally, this study also revealed a relationship between the anti-commensal response and richness of fungal species or strains within a genus showing that intense IgG responses to pathobionts, such as *Malassezia* and *Candida* (for the budding and invasive form), were significantly associated with lower intra-genus richness. Species within the same genus with different virulent capabilities or dominance of a more virulent species in the same genera may be responsible for the lowest inter-genus diversity and the intense immune response. In the present study, healthy donors with lower intra-genus richness (ASV ≤ 2) were mainly colonized by *C*. *albicans* and *M*. *restricta*. Evidences from in vitro studies and animal models proposed that the human immune system has learnt to differentiate fungal antigens from *Candida* hypha and yeast, tolerating those from the sporulated form but reacting to hyphae antigens associated with invasion [47–49]. Our results confirmed these observations in humans and let us to hypothesize that immunoregulation processes might have an impact on gut fungal fitness.

Altogether, these results let us to propose that in healthy donors’ intestinal mycobiota influence anti-fungal antibody responses. Whereas most of the present findings concerning gut mycobiota composition agree with the existence of a core mycobiome [38], a high degree of inter- and intra-volunteer variability has been observed. In the present study, combining the results obtained from immunological and phylogenetic methods, we bring new evidence supporting the hypothesis that different fungal ecosystems exist and that they are associated with a distinct immunological fingerprint.

## Conclusions

Fungi-Flow method allows an easy and reliable measure of personalized humoral responses against commensal fungi. Combining sequencing technology with our novel Fungi-Flow immunological method, we propose that there are ecosystems in the human gut mycobiome associated with systemic humoral responses. Fungi-Flow will improve our knowledge concerning the role of IgG in immune and mycobiome homeostasis or as a marker to characterize immune responses related to autoimmune diseases. This new method could also represent an important advance in the diagnosis of emerging fungal infections for which there are presently no diagnostic methods [50, 51]

## Supplementary information

**Additional file 1: Figure S1**. Fungus budding *in vitro* conditions. A) Side scatter (SSC) and forward scatter (FSC) structural analysis of sporulated and budding forms of *C. albicans* and *A. fumigatus* after overnight (O/N) culture using different starting concentrations of yeast or spores/ml. B) Percentage of yeast and budding forms for each condition tested after O/N culture.

**Additional file 2: Figure S2**. Fungi-Flow optimal conditions and specificity for IgA assessment. A-B) Dose response relationship was evaluated by plotting median fluorescence intensity (MFI) obtained for IgA responses at different concentrations of *C. albicans* and *A. fumigatus* and serum antibody concentrations. Serum was obtained from patients with a confirmed hepatosplenic candidosis (HSC) or aspergilloma (AG). C-D) Specificity of IgA in sera from HSC, AG patients and healthy donor (HD). Results correspond to the mean and standard deviation (n=3). Statistical analysis was performed using Student’s t-test (* p< 0.5** *p< 0.05*, *** *p< 0.005*). E-F) IgA responses from breastmilk and bronchoalveolar lavage (BAL) samples of different donors (green and orange lines respectively) were tested for antibody binding to *C. albicans* and *A. fumigatus.* Controls: None = unstained fungal forms (gray filled histogram), isotype control (black line), IgA deficient serum (blue line).

**Additional file 3: Figure S3**. Fungal stock cryopreservation. A) Histograms show the effect of two cryopreservation mediums A and B on IgG binding in yeast and buddings forms of *C. albicans* compared with that obtained with freshly grown fungi. Effects of fungus cryopreservation on IgG binding was evaluated using a serum from a patient suffering from hepatosplenic candidiasis (HSC) and a pool of intravenous IgG (IVIG). The effect of congelation mediums was also measured in the different negative controls used. None = unstained fungal forms. NRA= non-relevant antibody, IC= Isotype control. B) Median fluorescence intensity (MFI) measured for each condition. Cryopreservation medium A contains: 1% (wt/vol) Bacto Peptone (BD Biosciences), 1% (wt/vol) Bacto yeast extract (BD Biosciences), 0.5% Glucose (wt/vol) (Sigma Aldrich) and 25% Glycerol (vol/vol) (Sigma Aldrich) in distilled water. Cryopreservation medium B contains: 10% of Glycerol (vol/vol) (Sigma Aldrich) in PBS (GIBCO) was also evaluated.

**Additional file 4: Figure S4**. Relationship between fungal gut abundance and anti-commensal IgG responses. A) Relative abundance of the fifty most abundant fungal genera in gut of healthy donors. B) Correlation between the fungal relative abundance in gut of healthy donors and the intensity of anti-commensal systemic IgG response for the nine more abundant intestinal fungi. Statistical analysis was performed using Spearman tests. Ns= non-significant difference.

**Additional file 5: Figure S5**. Fungal burden in healthy donors. 16S or 18S ribosomal RNA gene levels were determined by real-time quantitative polymerase chain reaction (qPCR). To avoid confounding factors (e.g. different stool hydration) 18S results were normalized using the bacterial 16S gene, as previously described [34]. The relative fungal load was calculated using the 2-(2(-∆∆Ct)) method. Global fungus level in healthy donors from ecosystem 1 and 2 showed no significant differences.

## Data Availability

All data generated or analyzed during this study are available.

## Abbreviations

ASCA: Anti-*Saccharomyces cerevisiae* antibodies
ASV: Amplicon sequence variant
Ig: Immunoglobulins
MFI: Median fluorescence intensity
