# Supplementary figures and images for: Systemic anti-commensal response to fungi analyzed by flow cytometry is related to gut mycobiome ecology
Source: Microbiome. 2020 Nov 15;8:159. doi: 10.1186/s40168-020-00924-8 (PMC7667786; doi:10.1186/s40168-020-00924-8)

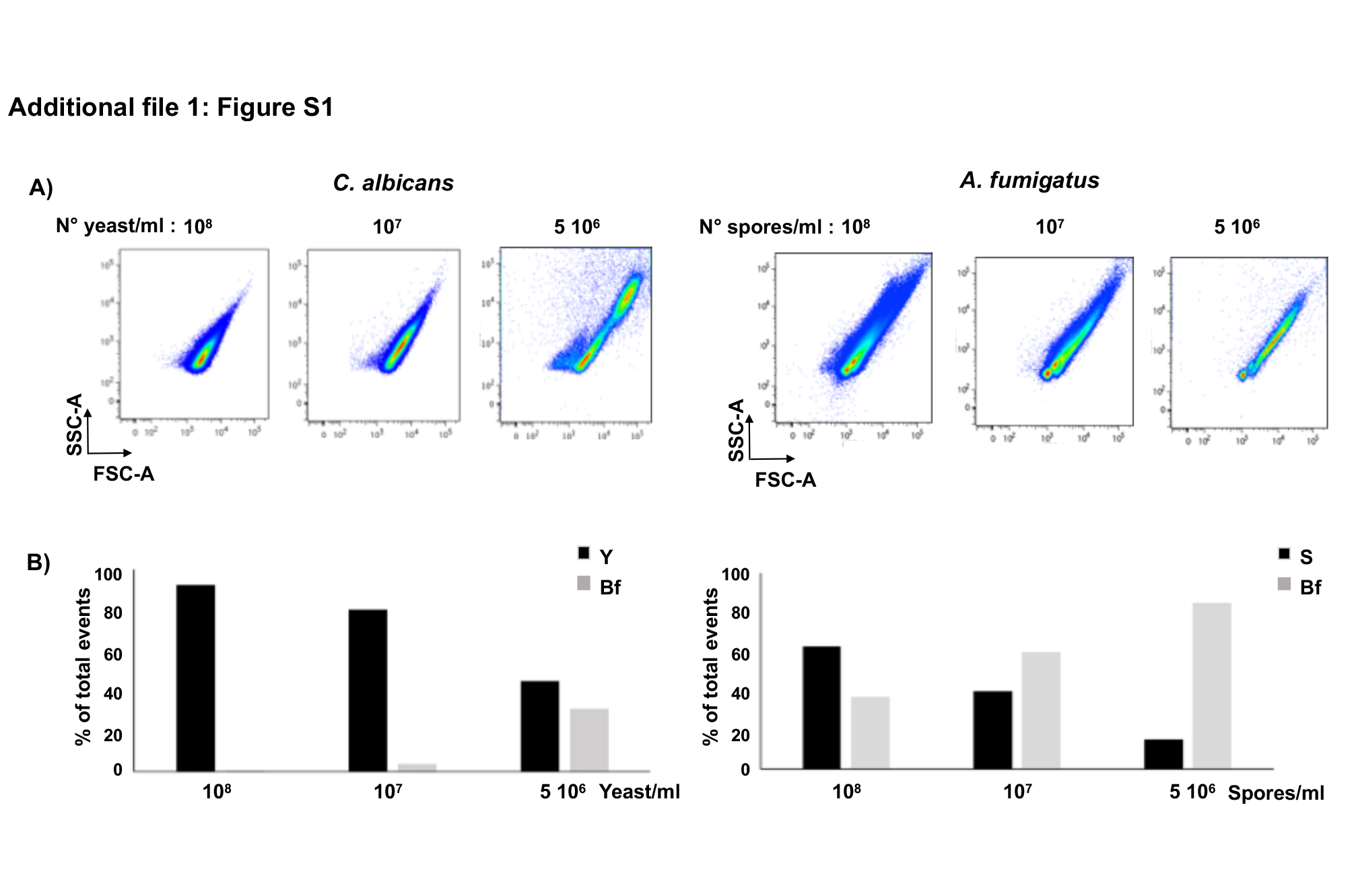

Supplement: Supplementary file 2 — Additional file 1: Figure S1. Fungus budding in vitro conditions. A) Side scatter (SSC) and forward scatter (FSC) structural analysis of sporulated and budding forms of C. albicans and A. fumigatus after overnight (O/N) culture using different starting concentrations of yeast or spores/ml. B) Percentage of yeast and budding forms for each condition tested after O/N culture. [file 40168_2020_924_MOESM1_ESM.tif]

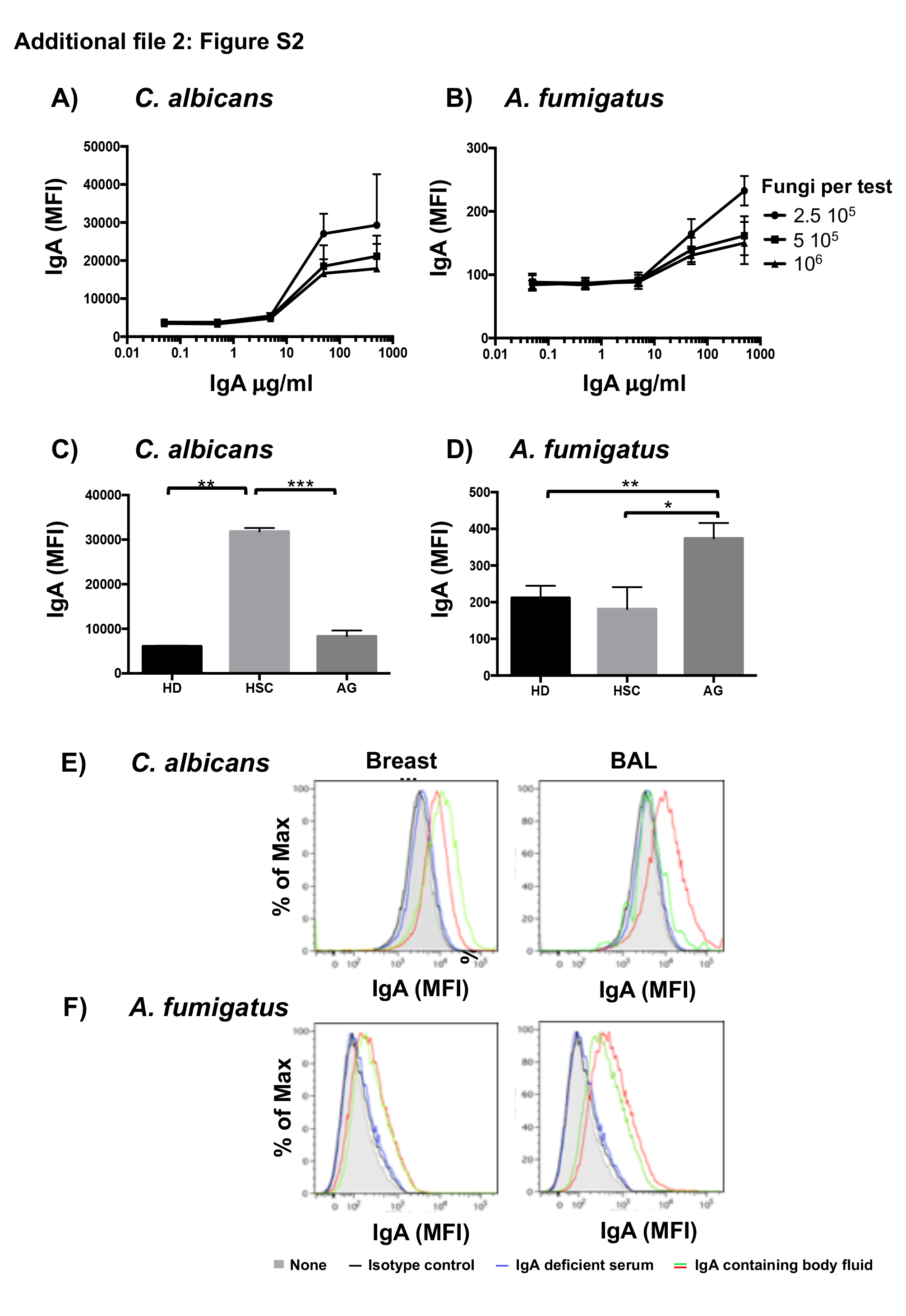

Supplement: Supplementary file 3 — Additional file 2: Figure S2. Fungi-Flow optimal conditions and specificity for IgA assessment. A-B) Dose response relationship was evaluated by plotting median fluorescence intensity (MFI) obtained for IgA responses at different concentrations of C. albicans and A. fumigatus and serum antibody concentrations. Serum was obtained from patients with a confirmed hepatosplenic candidosis (HSC) or aspergilloma (AG). C-D) Specificity of IgA in sera from HSC, AG patients and healthy donor (HD). Results correspond to the mean and standard deviation (n=3). Statistical analysis was performed using Student’s t-test (* p< 0.5** p< 0.05, *** p< 0.005). E-F) IgA responses from breastmilk and bronchoalveolar lavage (BAL) samples of different donors (green and orange lines respectively) were tested for antibody binding to C. albicans and A. fumigatus. Controls: None = unstained fungal forms (gray filled histogram), isotype control (black line), IgA deficient serum (blue line). [file 40168_2020_924_MOESM2_ESM.tif]

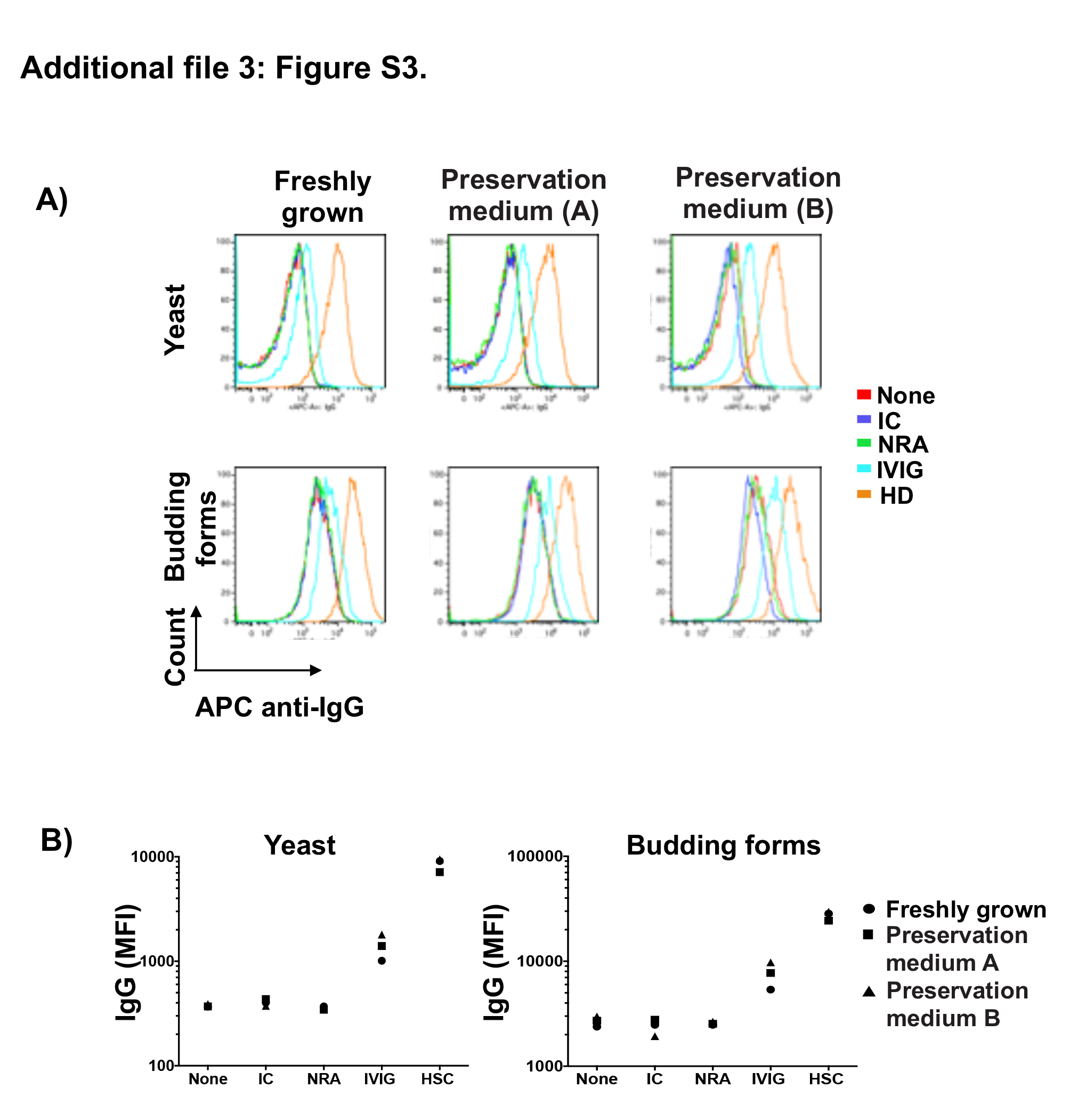

Supplement: Supplementary file 4 — Additional file 3: Figure S3. Fungal stock cryopreservation. A) Histograms show the effect of two cryopreservation mediums A and B on IgG binding in yeast and buddings forms of C. albicans compared with that obtained with freshly grown fungi. Effects of fungus cryopreservation on IgG binding was evaluated using a serum from a patient suffering from hepatosplenic candidiasis (HSC) and a pool of intravenous IgG (IVIG). The effect of congelation mediums was also measured in the different negative controls used. None = unstained fungal forms. NRA= non-relevant antibody, IC= Isotype control. B) Median fluorescence intensity (MFI) measured for each condition. Cryopreservation medium A contains: 1% (wt/vol) Bacto Peptone (BD Biosciences), 1% (wt/vol) Bacto yeast extract (BD Biosciences), 0.5% Glucose (wt/vol) (Sigma Aldrich) and 25% Glycerol (vol/vol) (Sigma Aldrich) in distilled water. Cryopreservation medium B contains: 10% of Glycerol (vol/vol) (Sigma Aldrich) in PBS (GIBCO) was also evaluated. [file 40168_2020_924_MOESM3_ESM.tif]

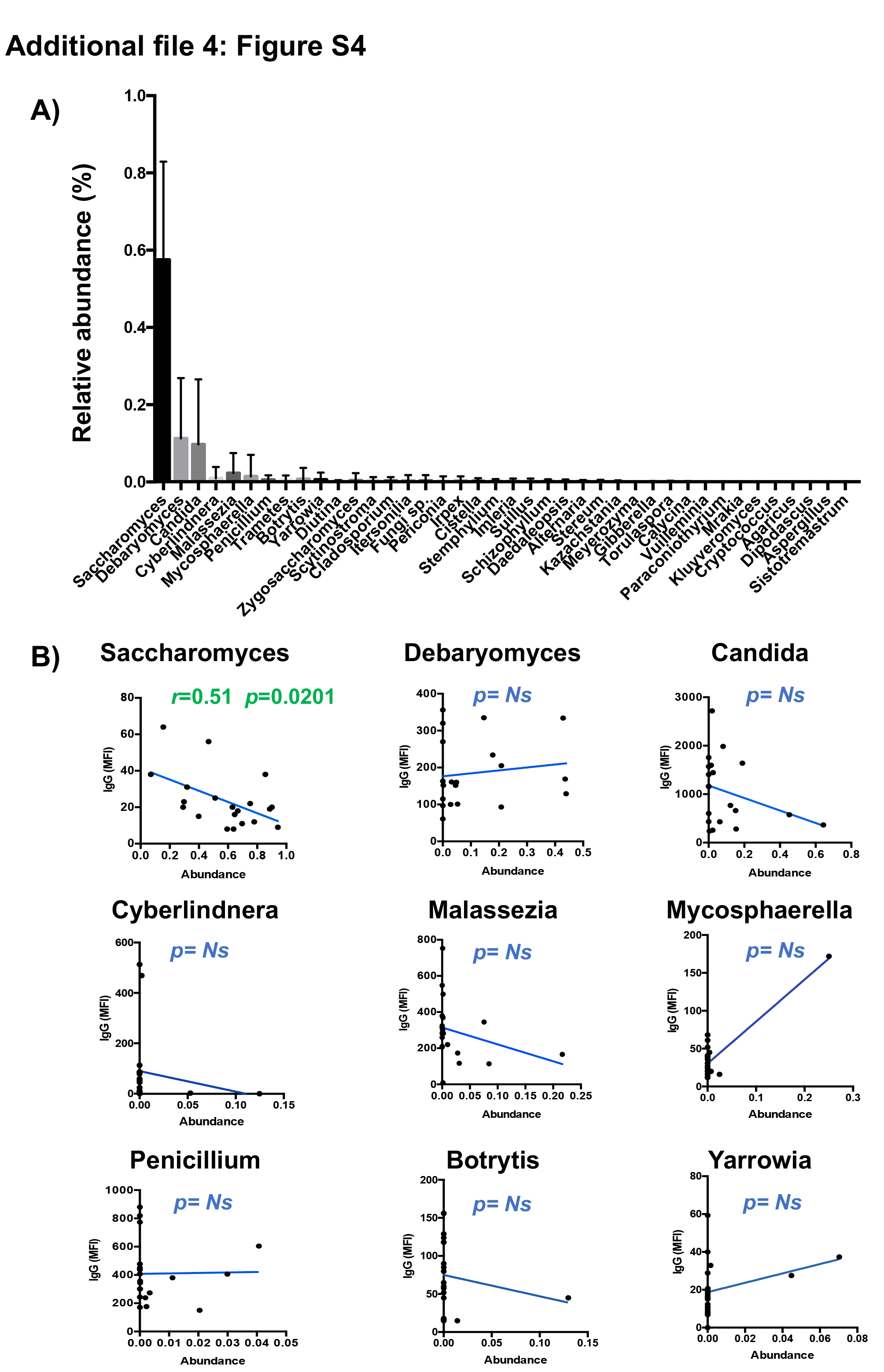

Supplement: Supplementary file 5 — Additional file 4: Figure S4. Relationship between fungal gut abundance and anti-commensal IgG responses. A) Relative abundance of the fifty most abundant fungal genera in gut of healthy donors. B) Correlation between the fungal relative abundance in gut of healthy donors and the intensity of anti-commensal systemic IgG response for the nine more abundant intestinal fungi. Statistical analysis was performed using Spearman tests. Ns= non-significant difference. [file 40168_2020_924_MOESM4_ESM.tif]

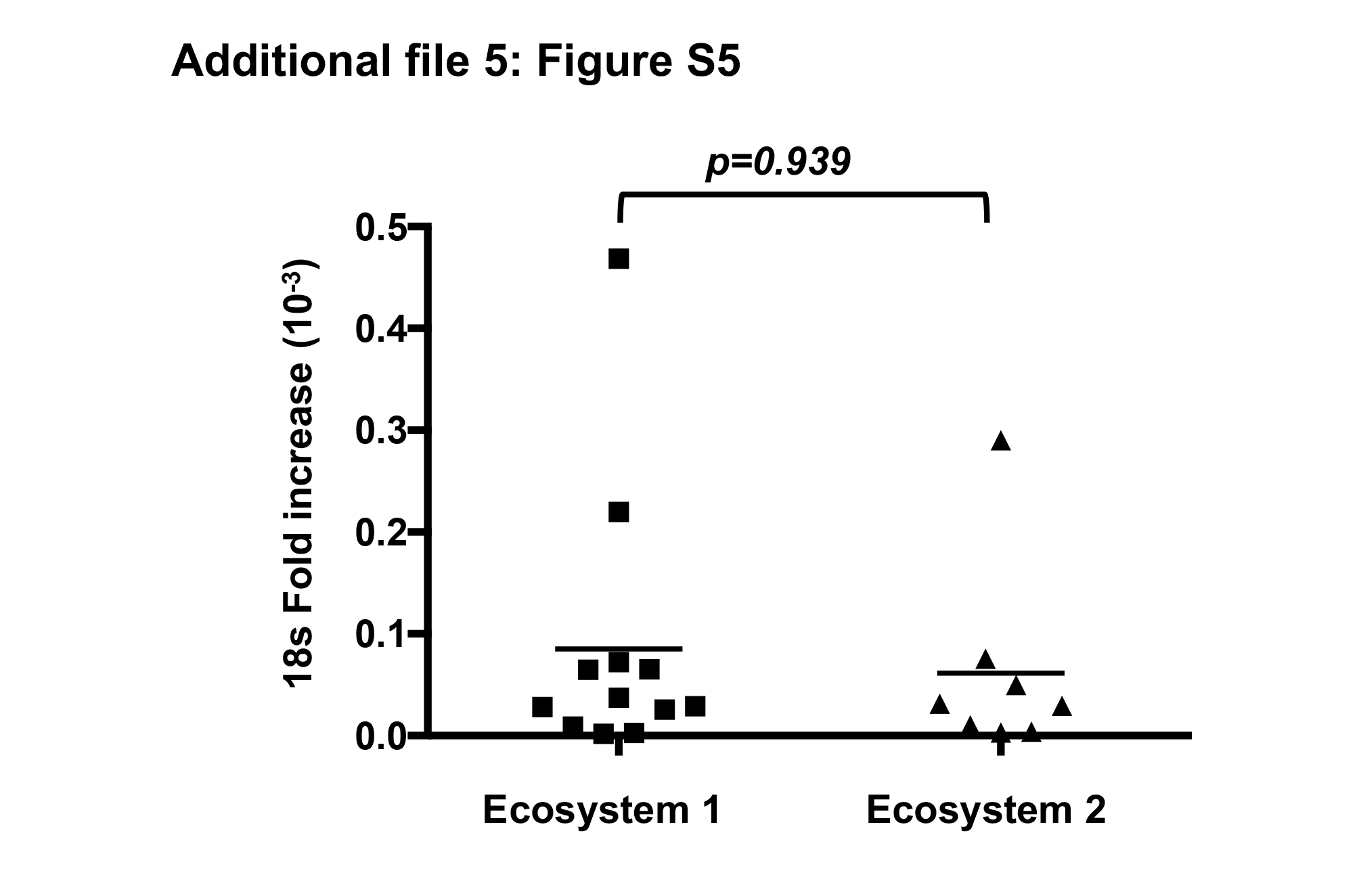

Supplement: Supplementary file 6 — Additional file 5: Figure S5. Fungal burden in healthy donors. 16S or 18S ribosomal RNA gene levels were determined by real-time quantitative polymerase chain reaction (qPCR). To avoid confounding factors (e.g. different stool hydration) 18S results were normalized using the bacterial 16S gene, as previously described [34]. The relative fungal load was calculated using the 2-(2(-∆∆Ct)) method. Global fungus level in healthy donors from ecosystem 1 and 2 showed no significant differences. [file 40168_2020_924_MOESM5_ESM.tif]
